# Supplementary material for: Widefield imaging of rapid pan-cortical voltage dynamics with an indicator evolved for one-photon microscopy
Source: Nat Commun. 2023 Oct 12;14:6423. doi: 10.1038/s41467-023-41975-3 (PMC10570354; doi:10.1038/s41467-023-41975-3)
Supplement: Supplementary file 1 — Supplementary Information [file 41467_2023_41975_MOESM1_ESM.pdf]

Supplementary Information for

Widefield imaging of rapid pan-cortical voltage dynamics with an indicator evolved for one-photon microscopy

Xiaoyu Lu[1,*], Yunmiao Wang[2,3,*], Zhuohe Liu[4], Yueyang Gou[5], Dieter Jaeger[3,#], François St-Pierre[1,4,5,6,#]

[1]Systems, Synthetic, and Physical Biology Program, Rice University, Houston, Texas, 77005, USA
[2]Neuroscience Graduate Program, Emory University, Atlanta, Georgia, 30322, USA
[3]Biology Department, Emory University, Atlanta, Georgia, 30322, USA
[4]Department of Electrical and Computer Engineering, Rice University, Houston, Texas, 77005, USA
[5]Department of Neuroscience, Baylor College of Medicine, Houston, Texas, 77030, USA
[6]Department of Biochemistry and Molecular Biology, Baylor College of Medicine, Houston, Texas, 77030, USA

[*] These authors have contributed equally to the work
[#] These authors have jointly supervised the work

Correspondence should be addressed to: Dieter Jaeger (djaeger@emory.edu) and François St-Pierre (stpierre@bcm.edu)

Contents

Supplementary Figure 1. Design and benchmarking of the high-throughput multiparametric one-photon screening platform, related to Figure 1. 2

Supplementary Figure 2. JEDI-1P's responses to voltage are modulated by pH, related to Figure 2. 3

Supplementary Figure 3. JEDI-1P provides more precise tracking of rapid spiking activity than ASAP2s and ASAP3, related to Figure 2. 4

Supplementary Figure 4. JEDI-1P displays a larger photon budget than ASAP2s and ASAP3, related to Figure 2 5

Supplementary Figure 5. JEDI-1P has larger response amplitude and brightness compared with JEDI-2P under one-photon excitation. 6

Supplementary Figure 6. While JEDI-1P displays response amplitude and brightness similar to JEDI-2P under two-photon excitation, its photostability is comparatively lower. 8

Supplementary Figure 7. JEDI-1P reports action potentials and subthreshold activities in dissociated neurons, related to Figure 2. 9

Supplementary Figure 8. Regression is best done stepwise and can be achieved using different red fluorescent proteins 10

Supplementary Figure 9. Example of preprocessing steps on pixel-wise time series. 11

Supplementary Figure 10. Simultaneous voltage imaging and LFP in awake resting animals demonstrate JEDI-1P's fast responses *in vivo* 12

Supplementary Figure 11. Widefield JEDI-1P-Kv imaging captures fast responses to single whisker deflections. 14

Supplementary Figure 12. Widefield voltage imaging of JEDI-1P-Kv captures oscillatory response up to 60 Hz. 15

References 16

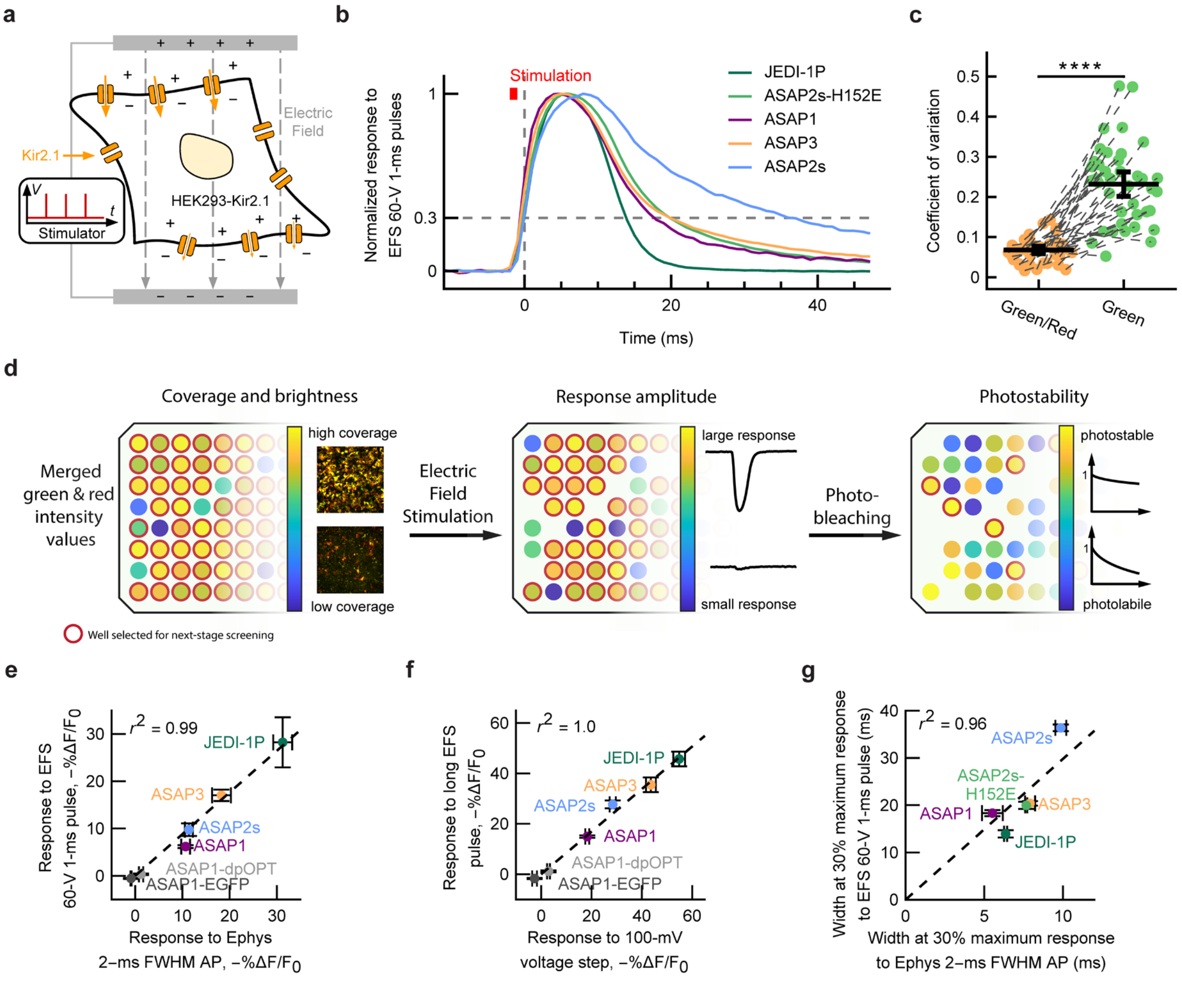

Supplementary Figure 1. Design and benchmarking of the high-throughput multiparametric one-photon screening platform, related to Figure 1.

a. Screening was conducted in HEK293 cells expressing Kir2.1 from the chromosome. Electric field stimulation (EFS) is thought to result in transient membrane hyperpolarization or depolarization, depending on the position of the membrane in the electrical field. Because Kir2.1 is inwardly rectifying, potassium inflows are larger than outflows, causing net depolarization.

b. 1-ms EFS produced transient depolarizations. Except for the slow indicator ASAP2s, other GEVIs reported these spikes with a fluorescence response lasting 10-20 ms, measured at 30% of the peak response.

c. To normalize brightness measurements for variations in expression level, we co-expressed GEVIs with the red fluorescent protein mCherry. Quantifying brightness as the ratio of GEVIs and mCherry produced a lower coefficient of variation than measuring brightness from GEVI fluorescence alone. $p = 1.15 \times 10^{-12}$ (paired t-test, two-tailed). n = 40 wells. Each data point is the coefficient of variation from 4 fields-of-view per well. In each field-of-view, green and red intensity values were normalized by the number of selected pixels. The green/red ratio in each field-of-view was computed by dividing the normalized green intensity value with the normalized red intensity value. Each well was independently transfected. Error bars denote 95% CI of the mean.

d. Our screen evaluated multiple performance metrics in most wells. Because wells with the lowest expression or brightness typically produced noisy results, we excluded these wells from our response amplitude screen. To maximize screening throughput, we did not evaluate photostability for constructs that did not respond to voltage.

e-g. Same as Figure 1d-f but with JEDI-1P included. n = 4 independent transfections (EFS) or 3 (ASAP1-dpOPT), 4 (ASAP1-EGFP), 4 (ASAP1), 10 (ASAP2s), 4 (ASAP2s-H152E), 8 (ASAP3) and 11 (JEDI-1P) HEK293A cells (voltage clamp) per GEVI. Error bars or shading denote 95% confidence interval (CI) of the mean.

  e. Fluorescence responses to 1-ms EFS pulses and AP-like waveforms (2-ms width at half-maximum, +30 mV peak, -70 mV baseline, whole-cell voltage clamp) are highly correlated.

  f. Fluorescence responses to EFS pulse trains and 1-s 100-mV step depolarizations (-70 mV to + 30 mV, whole-cell voltage clamp) are highly correlated.

  g. Optical response widths to 1-ms EFS pulses and AP-like waveforms are highly correlated. Widths were measured at 30% of the response peak.

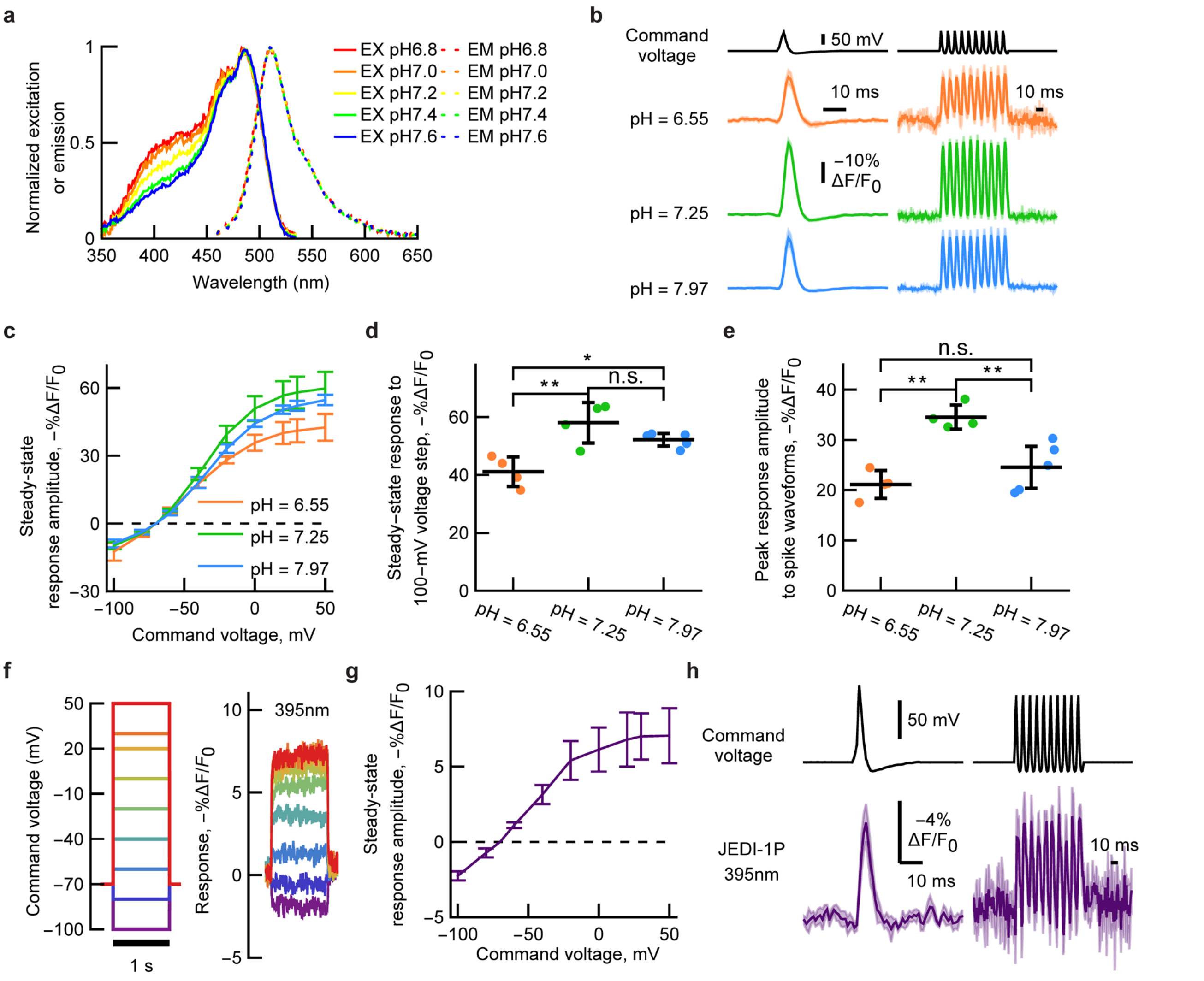

Supplementary Figure 2. JEDI-1P's responses to voltage are modulated by pH, related to Figure 2.

a. The excitation spectrum of JEDI-1P varies with the pH, with a higher 400-nm shoulder at lower pH. The emission spectrum of JEDI-1P does not vary with pH. n = 3 (pH = 6.8), n = 3 (pH = 7.0), n = 4 (pH = 7.2), n = 5 (pH = 7.4), n = 6 (pH = 7.6) independent transfections in HEK293-Kir2.1 cells.

b-e. JEDI-1P responds maximally at physiological pH. n = 4 (pH = 6.55), n = 4 (pH = 7.25) and n = 5 (pH = 7.97) HEK293A cells.

- b. Mean response to spike waveforms as described in Fig. 2d.
- c. Steady-state fluorescence versus voltage curves at different pH values. $p = 0.0009$ (Two-way ANOVA).
- d. JEDI-1P has a larger response to 100-mV step voltages (−70 mV to +30 mV) at physiological pH than at more acidic pH. $p = 0.0026$ (One-way ANOVA).
- e. JEDI-1P has a larger peak response amplitude to single AP waveforms at physiological pH than at more acidic or basic pH. $p = 0.001$ (One-way ANOVA).

f-h. JEDI-1P displays small bright-to-dim responses at 395nm excitation. n = 6 (f-g) and n = 5 (h) HEK293A cells.

- f. Mean response amplitude to 1-s voltage steps.
- g. Quantification of (f).
- h. *Left*: mean response to spike waveforms simulating APs (2-ms full-width at half-maximum, +30 mV peak, baseline at –70 mV). *Right*: mean response to a 100-Hz spike train waveform.

All panels: **** $p < 0.0001$; *** $p < 0.001$; ** $p < 0.01$; * $p < 0.05$; n.s. $p > 0.05$. Unless otherwise noted, one-way ANOVA followed by Tukey's multiple comparison test. Error bars or shading denote 95% confidence interval (CI) of the mean.

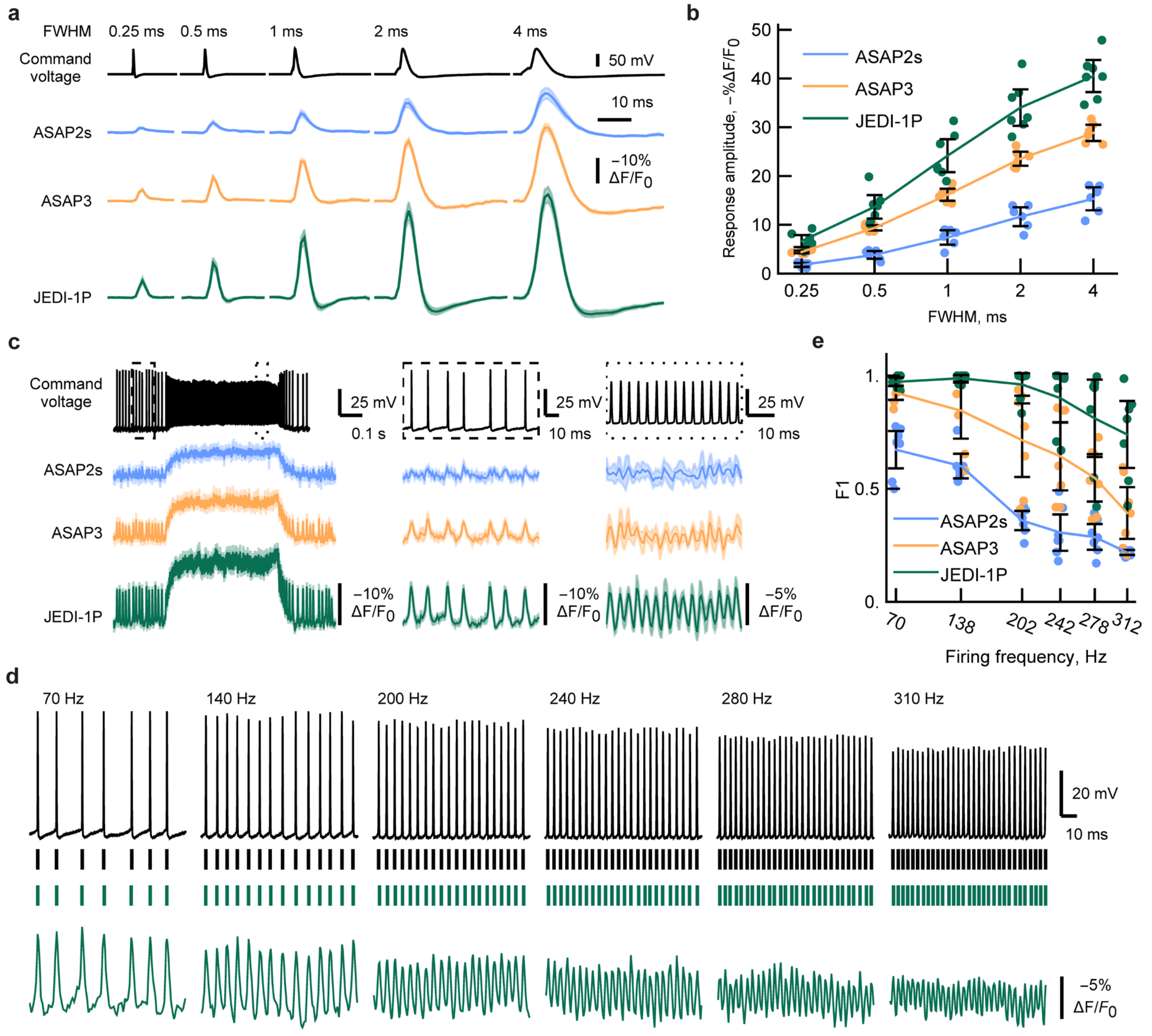

Supplementary Figure 3. JEDI-1P provides more precise tracking of rapid spiking activity than ASAP2s and ASAP3, related to Figure 2.

a-b. JEDI-1P shows larger responses to simulated action potentials of different widths ($p = 2.40 \times 10^{-8}$, Two-way ANOVA; Post-hoc comparisons in Supplementary Statistics). Fluorescence traces (a) and quantification (b) are shown. Waveforms have the following characteristic: 0.25~4-ms FWHM (full width at half maximum), +30 mV peak, baseline at –70 mV. n = 6 (ASAP2s), n = 6 (ASAP3), and n = 7 (JEDI-1P) HEK293A cells.

c-e. JEDI-1P follows narrow, high-frequency action potential waveforms better than ASAP2s and ASAP3. Waveforms were recorded from Purkinje cells in brain slices (see methods) with the following characteristic: 0.26~0.51-ms, 33~60-mV amplitude. n = 7 HEK293A cells per variant.

c. Mean fluorescence responses. Boxes indicate zoom-in views of the membrane potential before (dashed, middle panel) and during (dotted, right panel) a 500-pA current injection.

d. JEDI-1P detects action potentials of up to 310 Hz in single trials. Representative 100-ms segments from traces acquired from a representative cell are shown with their corresponding spiking frequency. Black and green bars are detected spikes from the electrophysiological and fluorescence traces, respectively (see Methods).

e. JEDI-1P detects the action potentials with higher accuracy (F1) than ASAP2s and JEDI3. $p = 5.58 \times 10^{-7}$ (Two-way ANOVA; Post-hoc comparisons in Supplementary Statistics).

All panels: Experiments were conducted at 32-35°C. The acquisition frequency was 1 kHz. Darker traces are the mean. Error bars and shading denote the 95% confidence interval.

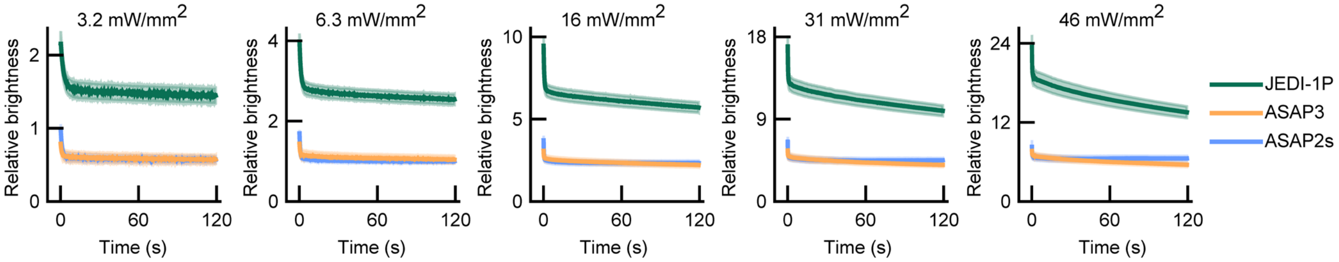

Supplementary Figure 4. JEDI-1P displays a larger photon budget than ASAP2s and ASAP3, related to Figure 2. 2-min fluorescence trace of JEDI-1P, ASAP2s, and ASAP3 illuminated with 470/24-nm light at an irradiance of 3.2 ~ 46 mW/mm^2 at the sample plane, normalized to the brightness of ASAP2s in the first frame. n = 12 (ASAP2s), n = 11 (ASAP3) and n = 12 (JEDI-1P) independent transfections per GEVI. Darker traces are the mean. Shaded areas denote the 95% confidence interval.

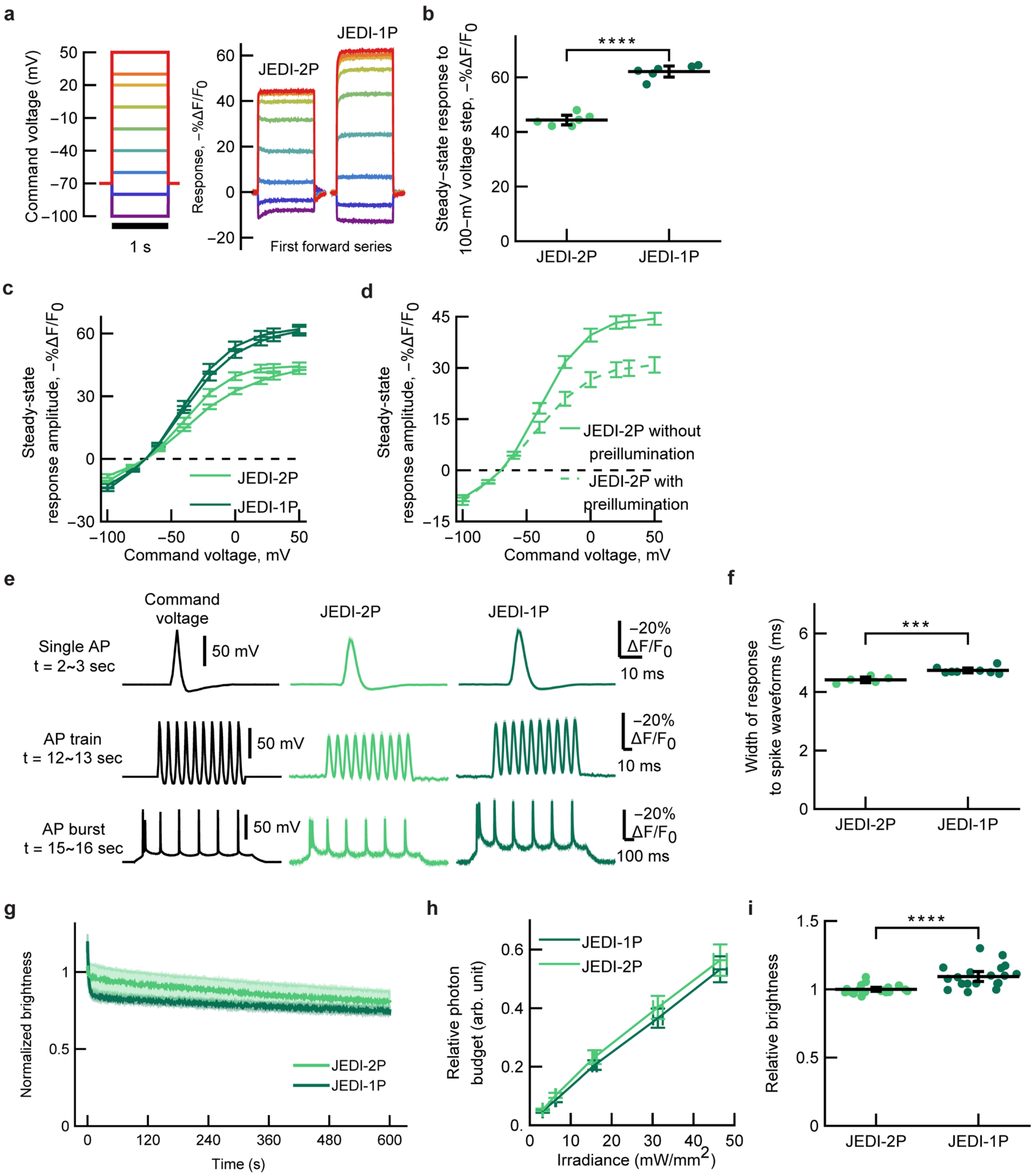

Supplementary Figure 5. JEDI-1P has larger response amplitude and brightness compared with JEDI-2P under one-photon excitation.

All data acquired using 470/24-nm excitation light.

a-d. Response amplitude to voltage steps under 1P illumination. n = 5 HEK293A cells per variant.

a. Mean response to voltage steps.
b. JEDI-1P shows larger responses to 100-mV voltage steps (−70 mV to +30 mV) than JEDI-2P. $p = 3.51\times10^{-7}$ (t-test, two-tailed).
c. JEDI-2P shows hysteresis under 1P illumination. Voltage steps were performed in ascending order (from -100 mV to +50 mV), then in descending order (from +50 mV to -100 mV). Cells were clamped at −70 mV between each step. p = 0.0735 for JEDI-1P and p = 0.0004 for JEDI-2P (Two-way ANOVA comparing responses to ascending and descending steps).

d. 25-26-s illumination of JEDI-2P resulted in smaller response amplitudes to voltage steps. $p = 2.60\times10^{-6}$ (Two-way ANOVA). Pre-illumination was performed while JEDI-2P was clamped at −70 mV, similar to the resting membrane potential of many cell types of cortical neurons.

e-f. JEDI-1P displayed larger responses to spike waveforms compared with JEDI-2P. n = 8 (JEDI-1P), n = 5 (JEDI-1P) HEK293A cells.

e. Mean response to artificial APs as described in Fig. 2d (Single AP and AP train) and methods (AP Burst).

f. JEDI-1P's response to spike waveforms is slightly larger than JEDI-2P's. $p = 0.0008$ (t-test, two-tailed).

g-h. JEDI-1P and JEDI-2P have similar photostability. n = 8 independent transfections in HEK293-Kir2.1 cells per variant.

g. Fluorescence traces of JEDI-1P and JEDI-2P normalized to the brightness of JEDI-2P of the first frame. The irradiance was 3.2 mW/mm^2 at the sample plane,

h. JEDI-1P and JEDI-2P have similar photon budgets over a >10-fold range of irradiance levels (3.2–47 mW/mm^2). $p = 0.55$ (3.2 mW/mm^2), $p = 0.23$ (6.3 mW/mm^2), $p = 0.24$ (16 mW/mm^2), $p = 0.55$ (31 mW/mm^2), $p = 0.55$ (47 mW/mm^2). (Mann-Whitney tests with Holm-Šídák correction, two-tailed).

i. JEDI-1P has higher one-photon brightness compared with JEDI-2P. Brightness evaluation is described in Figure 1h. n = 4 independent transfections per variant, with 5 fields-of-view imaged per transfection. Data were normalized to the mean brightness of JEDI-2P. $p = 8.28\times10^{-5}$ (Welch's t-test, two-tailed).

All panels: **** $p < 0.0001$; *** $p < 0.001$; ** $p < 0.01$; * $p < 0.05$; n.s. $p > 0.05$. Unless otherwise noted, Unpaired t-test was used. Error bars or shading denote 95% confidence interval (CI) of the mean.

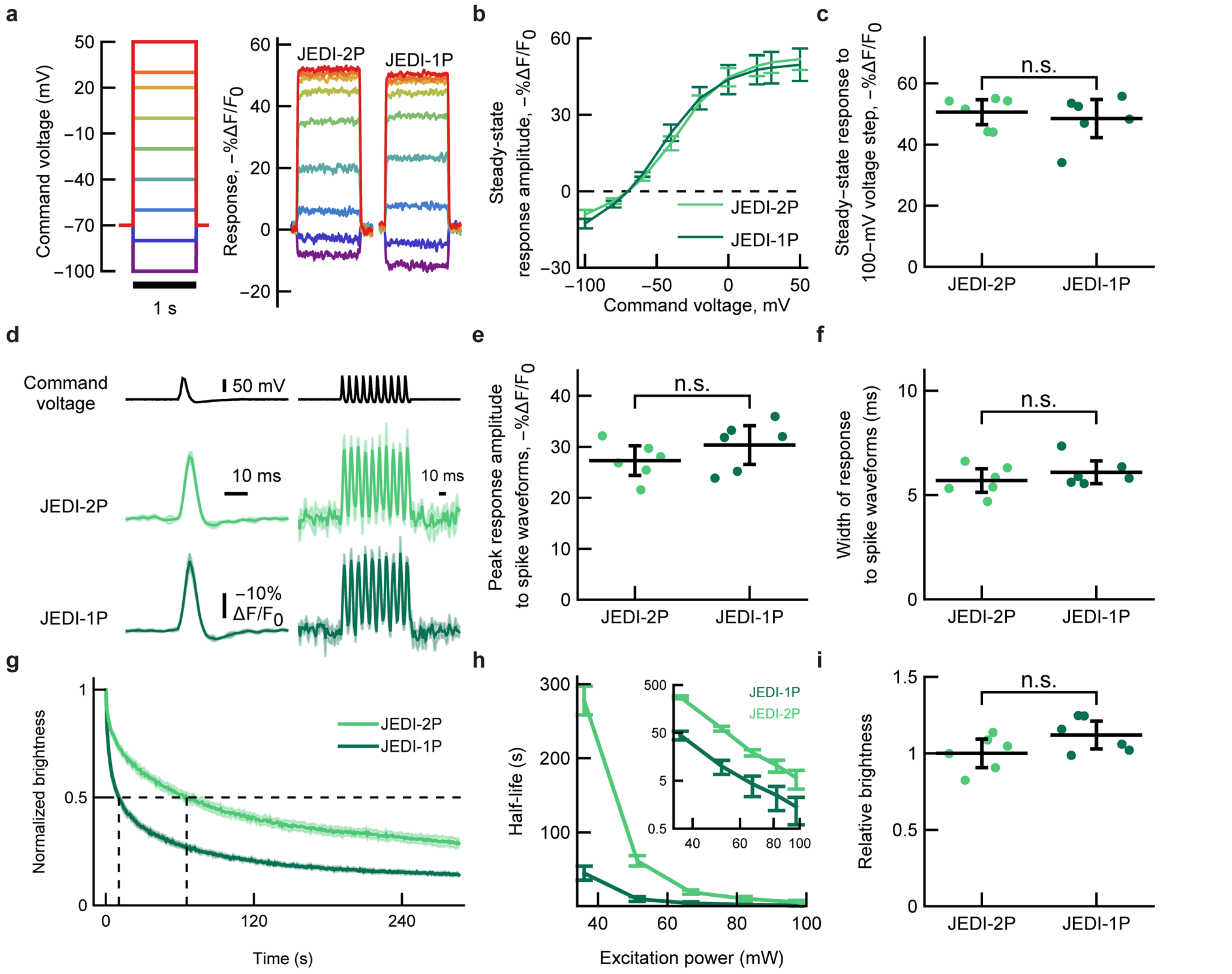

Supplementary Figure 6. While JEDI-1P displays response amplitude and brightness similar to JEDI-2P under two-photon excitation, its photostability is comparatively lower.

All data was acquired using 920-nm excitation light with resonant scanning at 440 Hz.

a-c. JEDI-1P has a similar steady-state response amplitude to 1-s step depolarizations compared to JEDI-2P. n = 6 HEK293A cells per variant.

a. Mean response to voltage steps. Fluorescence traces shown were smoothed by a 23-ms (10-frame) moving average.
b. Quantification of (a). JEDI-1P and JEDI-2P do not show statistically different response amplitudes (p = 0.8208, Two-way ANOVA). Adjusted p-value > 0.05 for all post-hoc comparisons (multiple unpaired t-test with Holm-Šídák correction, two-tailed).
c. Responses to 100-mV step voltages (−70 mV to +30 mV). p = 0.6017 (t-test, two-tailed).

d-f. JEDI-1P produces similar responses to spike waveforms as JEDI-2P. n = 6 HEK293A cells per variant.

d. Mean response to spike waveforms as described in Fig. 2d.
e. JEDI-1P has a similar peak response amplitude to single AP waveforms as JEDI-2P. p = 0.2406 (t-test, two-tailed).
f. JEDI-1P and JEDI-2P display responses of similar widths. p = 0.3437 (t-test, two-tailed).

g-h. JEDI-1P is less photostable than JEDI-2P. Photostability under 2P excitation was evaluated as described previously[1]. n = 8 independent transfections in HEK293-Kir2.1 cells per variant.

g. Normalized mean fluorescence time course. Dashed lines indicate half-lives.
h. Fluorescence half-lives of GEVIs as a function of the excitation power. The inset presents the data using a logarithmic scale. Power was measured at the sample plane.

i. JEDI-1P has a similar two-photon brightness as JEDI-2P. Two-photon brightness was evaluated as previously described[1]. n = 6 independent transfections in HEK293-Kir2.1 cells per variant. Data were normalized to the mean brightness of JEDI-2P. p = 0.102 (t-test, two-tailed).

All panels: Center lines or darker lines denote the mean. Error bars and shading denote the 95% confidence interval (CI) of the mean.

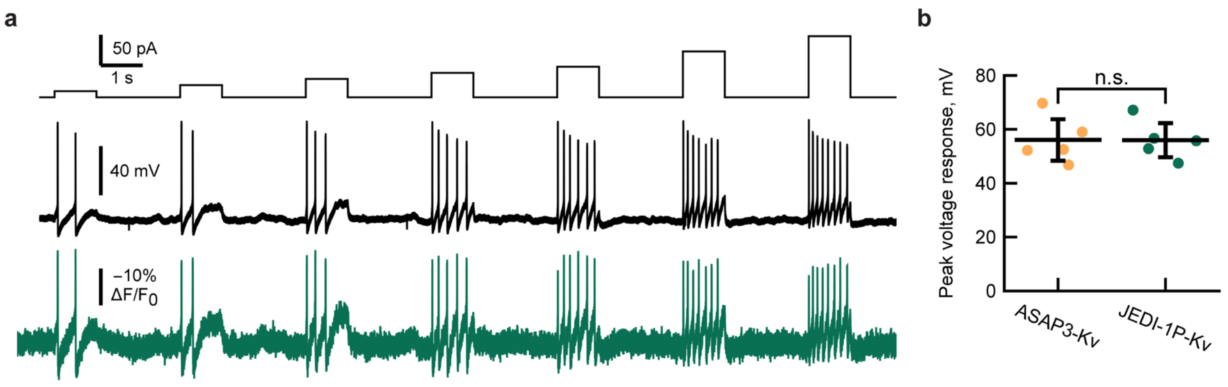

Supplementary Figure 7. JEDI-1P reports action potentials and subthreshold activities in dissociated neurons, related to Figure 2.

a. JEDI-1P-Kv reports action potentials and subthreshold activities in dissociated DIV12 cortical neurons in single trials. *Upper*: current injection protocol. *Middle*: membrane voltage recording from whole-cell current clamp. *Lower*: voltage imaging of JEDI-1P. Experiments were done at room temperature.

b. The amplitudes of the voltage responses used to compare JEDI-2P-Kv and ASAP3-Kv in Fig 2m are not significantly different. p = 0.9878 (t-test, two-tailed). n = 5 neurons per variant. Error bars and shading denote 95% confidence interval (CI) of the mean.

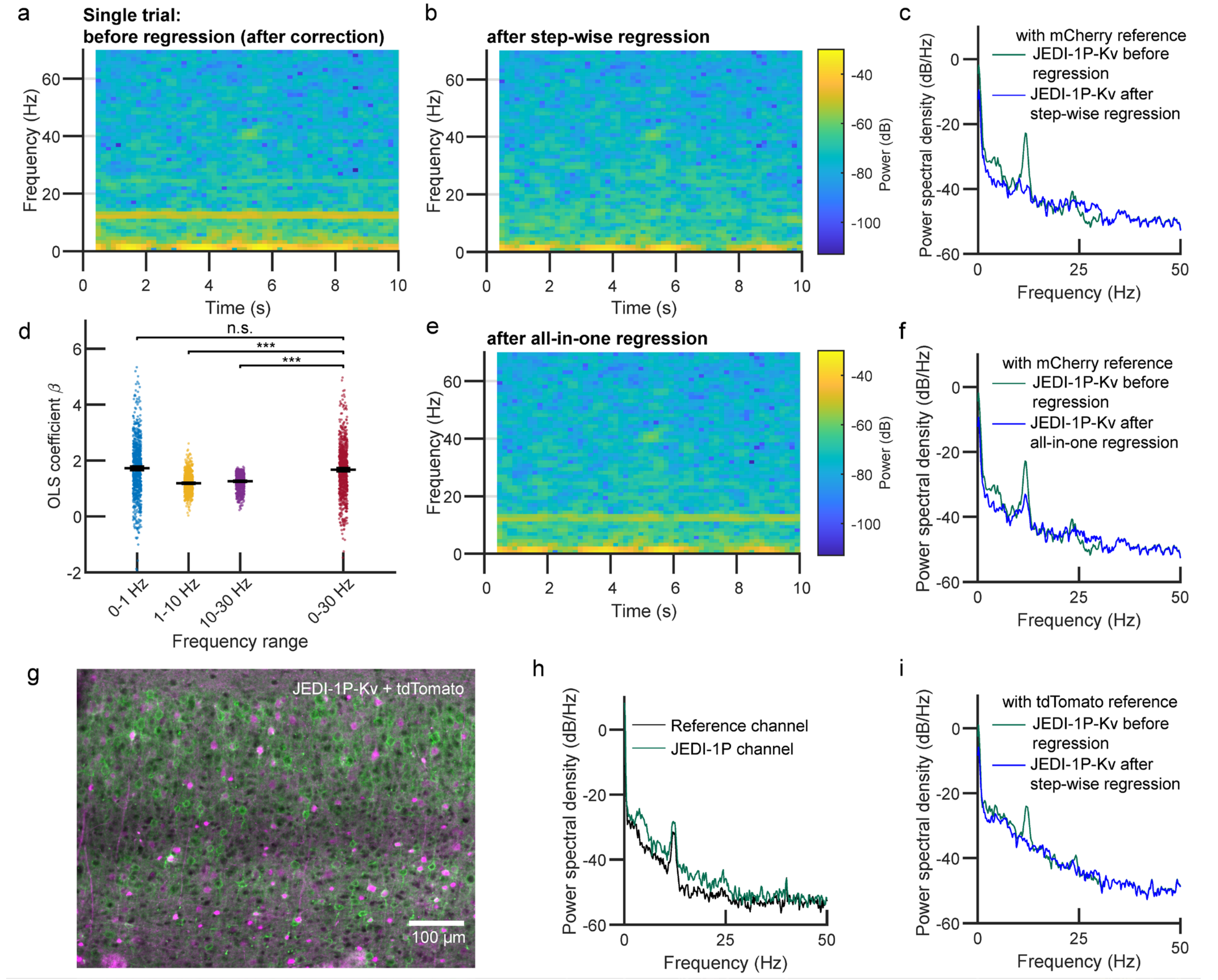

Supplementary Figure 8. Regression is best done stepwise and can be achieved using different red fluorescent proteins.

a-f. Stepwise regression stepwise removes hemodynamic and motion artifacts more efficiently than all-in-one regression. Data from awake mice.

- a-b. Single-trial JEDI-1P-Kv spectrograms of neural activity from an awake behaving animal before (a) and after (b) regression illustrates that stepwise regression can remove fast hemodynamic noise. (a) and (b) share the same color bar.
- c. Most fast hemodynamic signals were removed from the voltage trace. n = 25 trials from a representative mouse.
- d. Noises from different frequency ranges have different amplitudes, reflected by significantly different scale factors (β) of Ordinary Least Squares (OLS) regression. Center lines denote mean β. Error bars denote the 95% CI. n = 900 trials, 150 trials/mouse. ***, $p < 0.0001$; n.s., $p > 0.05$. Two-sided Mann-Whitney U test, p = 0.1710, 7.94×10^{-52}, and 1.95×10^{-41}.
- e. Computing all-in-one regression using a β value calculated from traces filtered with a broad frequency range retained significant artifacts at ~12 and ~24 Hz (yellow or high-dB horizontal bands). Same trial as (a) and (b), but with $j = g - \beta r$, where r was lowpass-filtered at 30 Hz.
- f. A representative all-in-step regression analysis retained significant fast hemodynamic noise, as shown by the significant peaks at ~12 Hz and their harmonics at ~24 Hz. n = 25 trials from the same mouse (same trials used in (c)).

g-i. tdTomato can be used as an alternative reference protein to mCherry.

- g. Sagittal slice of the cortex shows expression of JEDI-1P-Kv and tdTomato.
- h. tdTomato can report hemodynamic noise, as shown by the clear peak at ~12 Hz. Data is the mean of n = 20 trials at the same ROI from a representative mouse.
- i. The regression pipeline removed hemodynamic artifacts in mice co-injected with JEDI-1P-Kv and tdTomato AAVs. Data is the mean of n = 20 trials from a representative mouse.

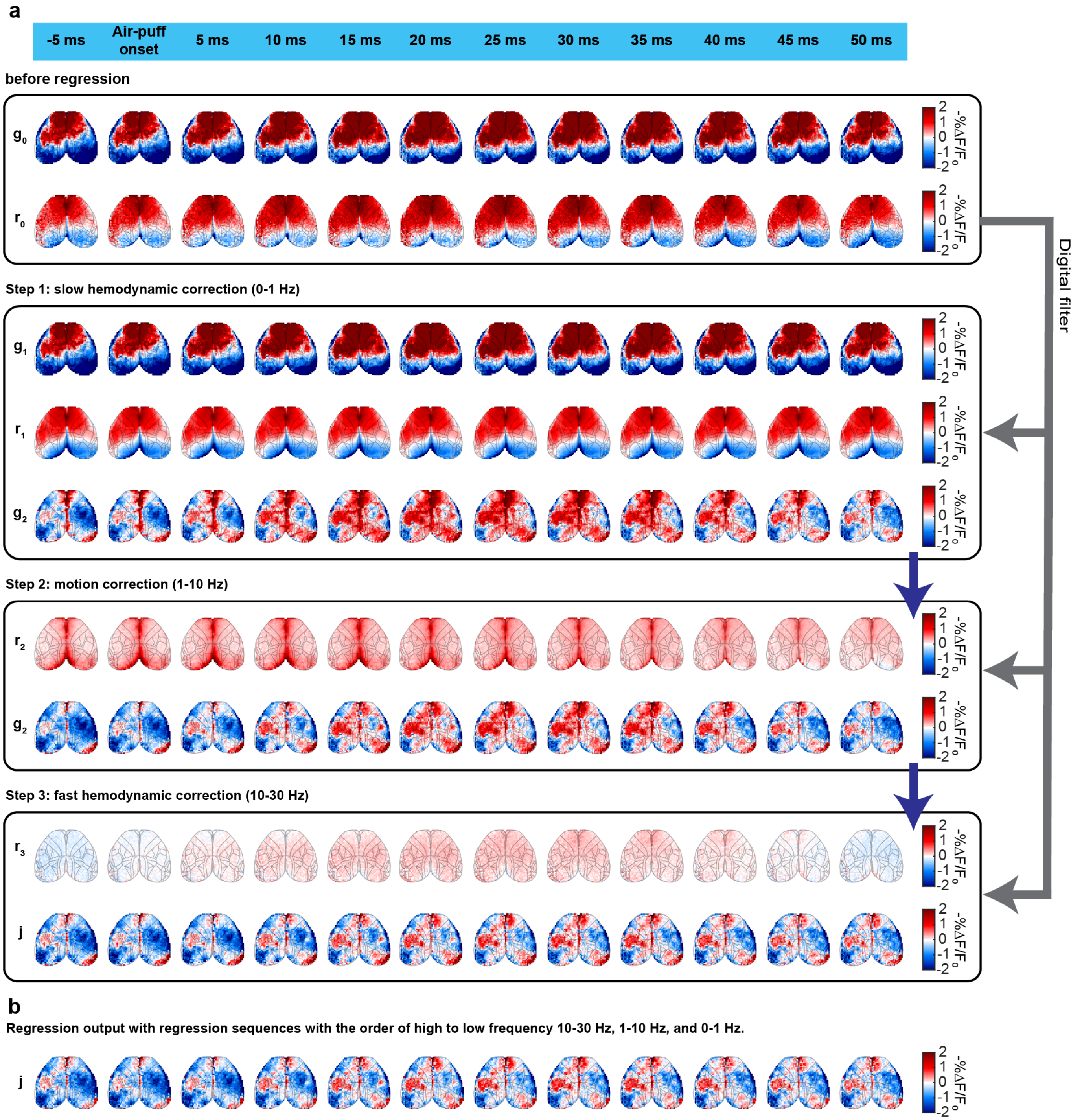

Supplementary Figure 9. Example of preprocessing steps on pixel-wise time series.

a. Each row shows JEDI-1P-Kv or mCherry reference signals at various steps during the regression workflow. Slow hemodynamic changes (0-1 Hz) dominated the signals in this example. For better visualization of 1-10 and 10-30 Hz regression steps, we thus regressed 0-1 Hz artifacts first. Regression steps are thus inverted compared with **Fig. 4**. Variable names as in **Fig. 4**.

b. Preprocessing pipeline output when carrying out the regression steps in the same order as in **Fig. 4**. The resulting spatial maps do not show obvious differences compared to those obtained in (a), when regression steps are inverted.

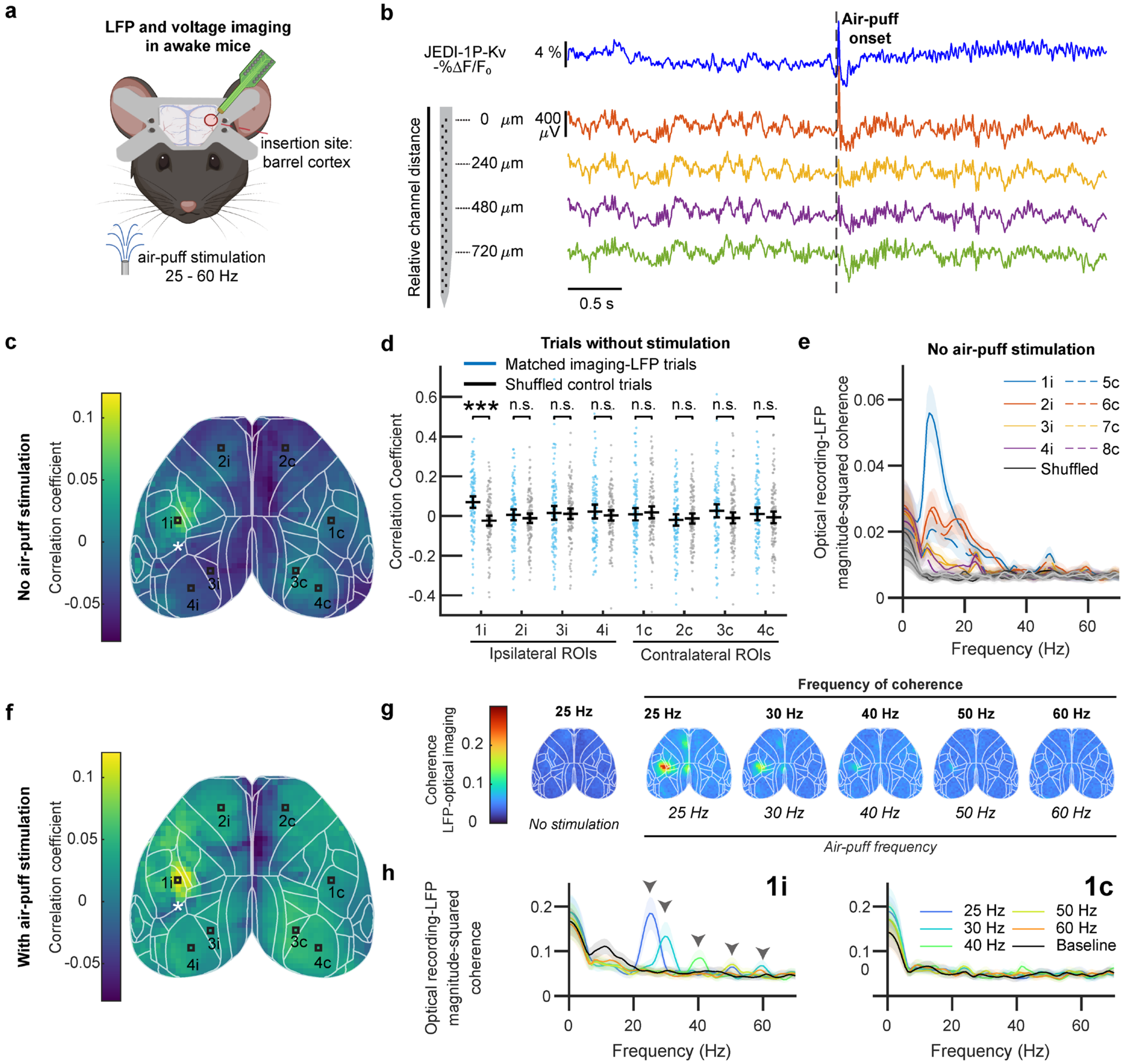

Supplementary Figure 10. Simultaneous voltage imaging and LFP in awake resting animals demonstrate JEDI-1P's fast responses *in vivo*.

a. Schematics of simultaneous optical and Local Field Potential (LFP) recordings with air-puff stimulation. The stimulation patterns are the same as in **Fig. 6j**. Each trial consisted of 4.5 s baseline and 3-s stimulation periods with 25, 30, 40, 50, 60 Hz air-puff stimulation.

b. JEDI-1P-Kv and LFPs show rapid responses to the onset of air-puff stimulation Voltage imaging was conducted close to the LFP recording site (ROI 1i in (c)). LFPs were recorded from 32 channels, with traces from 4 channels shown here (channels 1, 9, 17, 25). Depths are relative to the top channel.

c-e. Optical and LFP recordings show local correlation and coherence near the recording site.

c. Map showing the mean correlation between the LFP recording and JEDI-1P-Kv traces from 2×2 pixel ROIs. The purple asterisk indicates the LFP electrode insertion site. n = 53 trials, 1 mouse.

d. Correlation coefficient between the LFP recording and selected 2×2-pixel ROIs. n = 112 trials from 2 mice (53 and 59 trials, respectively). The horizontal lines denote the mean value. The error bars denote 95% confidence intervals. Two-sided Mann-Whitney U test, $p = 2.52 \times 10^{-6}$, 0.33, 0.77, 0.59, 0.38, 0.89, 0.07, and 0.33.e. Coherence between the LFP recording and selected ROIs. Same trials as (d). Error bands denote the 95% CI.

f-h. Optical and LFP recordings show coherence at the stimulation frequency.

f. Map showing the mean correlation (same as c) but for trials with air-puff stimulation. n = 51 trials.
g. Coherence between LFP recording from a single channel and all binned pixels of awake state trials with air-puff stimulations. n = 114 trials from the same 2 mice as (d) (51 and 63 trials for mice 1 and 2, respectively). The top row shows baseline coherence at 25 Hz without stimulation. The bottom row shows coherence maps at the same frequency as the stimulation. Each stimulation period was 3-s long.
h. Coherence between the LFP recording and ROI 1i (top) and 1c (bottom) at the same frequency as the stimulation. The LFP data (1i) show coherence at 60 Hz during the 60-Hz stimulation. Same trials as (g). LFP data in this analysis did not go through a 60-Hz notch filter, so coherence at this frequency could be measured. Error bands denote the 95% CI.

The mouse image in panel a was created using Biorender.com.

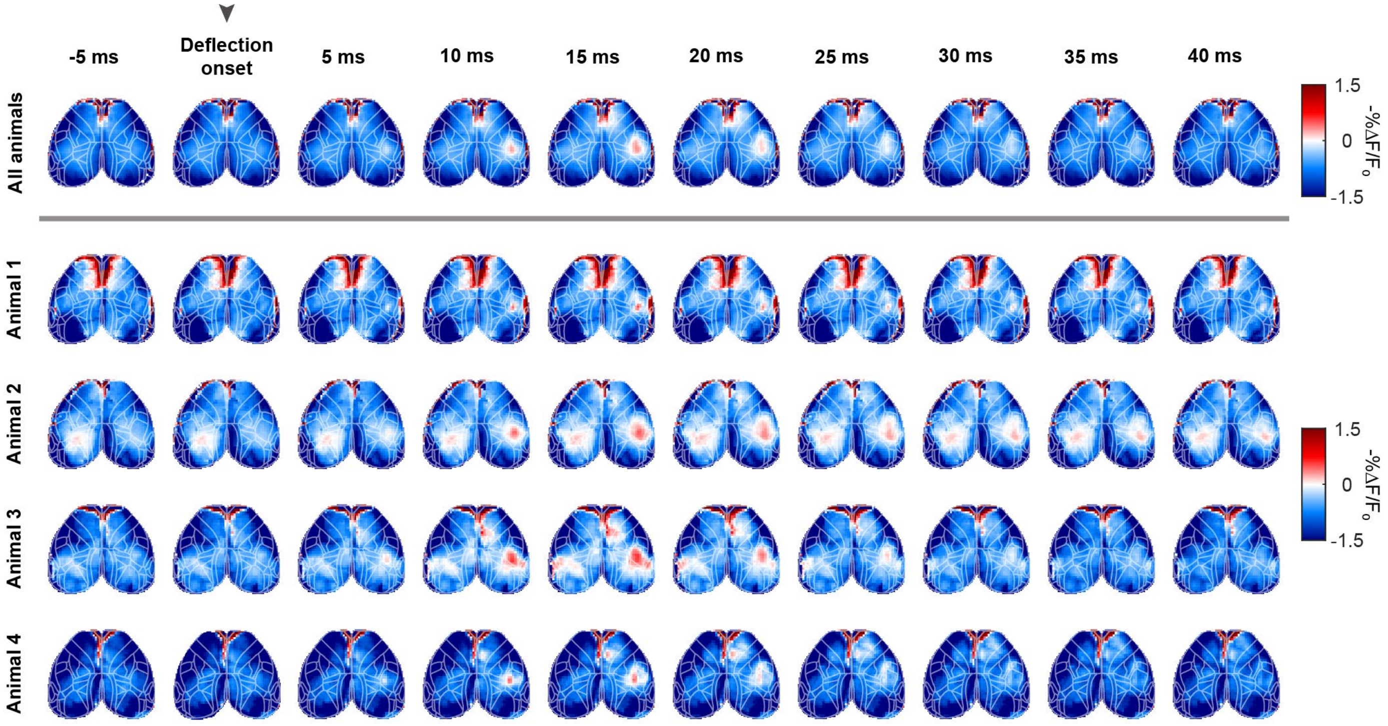

Supplementary Figure 11. Widefield JEDI-1P-Kv imaging captures fast responses to single whisker deflections. The top row shows the average response to single whisker deflections from 5 mice (n = 150 deflections/animal). The subsequent rows show trial-averaged responses from four individual animals. Responses from the fifth mouse are shown in **Fig. 6b**.

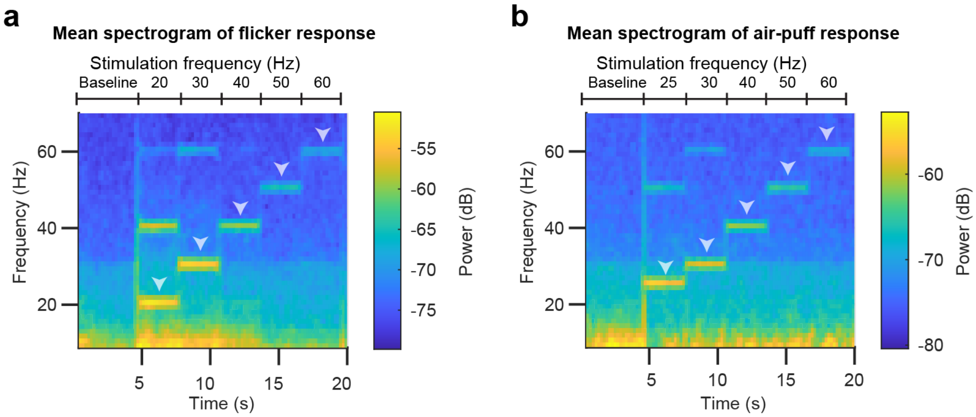

Supplementary Figure 12. Widefield voltage imaging of JEDI-1P-Kv captures oscillatory response up to 60 Hz.

a. Mean spectrogram of flicker responses from a 2×2-pixel ROI in V1, n = 4 mice, 10 trials/animal.

b. Mean spectrogram of air-puff responses from a 2×2-pixel ROI in the barrel cortex, n = 5 mice, 10 trials/animal.

Supplementary references

1. Liu, Z. *et al.* Sustained deep-tissue voltage recording using a fast indicator evolved for two-photon microscopy. *Cell* **185**, 3408-3425.e29 (2022).
