# Supplementary material for: Widefield imaging of rapid pan-cortical voltage dynamics with an indicator evolved for one-photon microscopy
Source: Nat Commun. 2023 Oct 12;14:6423. doi: 10.1038/s41467-023-41975-3 (PMC10570354; doi:10.1038/s41467-023-41975-3)
Supplement: Supplementary file 3 — Reporting Summary [file 41467_2023_41975_MOESM3_ESM.pdf]

nature portfolio

Corresponding author(s): François St-Pierre, Dieter Jaeger

Last updated by author(s): Aug 3, 2023

Reporting Summary

Nature Portfolio wishes to improve the reproducibility of the work that we publish. This form provides structure for consistency and transparency in reporting. For further information on Nature Portfolio policies, see our Editorial Policies and the Editorial Policy Checklist.

Statistics

For all statistical analyses, confirm that the following items are present in the figure legend, table legend, main text, or Methods section.

n/a	Confirmed	
☐	☒	The exact sample size (*n*) for each experimental group/condition, given as a discrete number and unit of measurement
☐	☒	A statement on whether measurements were taken from distinct samples or whether the same sample was measured repeatedly
☐	☒	The statistical test(s) used AND whether they are one- or two-sided<br>*Only common tests should be described solely by name; describe more complex techniques in the Methods section.*
☒	☐	A description of all covariates tested
☐	☒	A description of any assumptions or corrections, such as tests of normality and adjustment for multiple comparisons
☐	☒	A full description of the statistical parameters including central tendency (e.g. means) or other basic estimates (e.g. regression coefficient) AND variation (e.g. standard deviation) or associated estimates of uncertainty (e.g. confidence intervals)
☐	☒	For null hypothesis testing, the test statistic (e.g. *F*, *t*, *r*) with confidence intervals, effect sizes, degrees of freedom and *P* value noted<br>*Give P values as exact values whenever suitable.*
☒	☐	For Bayesian analysis, information on the choice of priors and Markov chain Monte Carlo settings
☒	☐	For hierarchical and complex designs, identification of the appropriate level for tests and full reporting of outcomes
☐	☒	Estimates of effect sizes (e.g. Cohen's *d*, Pearson's *r*), indicating how they were calculated

Our web collection on statistics for biologists contains articles on many of the points above.

Software and code

Policy information about availability of computer code

Data collection	NIS-Elements AR 4.6, LabVIEW NXG 5.1, MultiClamp 700B, Clampex 11.1, Zen 2.3 SP1 FP3 (black edition), Ultima_USB_dual-ver1207, RHD2000interface, MATLAB2021a, Bpod_Gen2
Data analysis	Zen 2.3 (blue edition), GraphPad Prism 9, MATLAB2021b, MATLAB2022a, MATLAB2022b, Wolfram Mathematica 13.

For manuscripts utilizing custom algorithms or software that are central to the research but not yet described in published literature, software must be made available to editors and reviewers. We strongly encourage code deposition in a community repository (e.g. GitHub). See the Nature Portfolio guidelines for submitting code & software for further information.

Data

Policy information about availability of data

All manuscripts must include a data availability statement. This statement should provide the following information, where applicable:

- Accession codes, unique identifiers, or web links for publicly available datasets
- A description of any restrictions on data availability
- For clinical datasets or third party data, please ensure that the statement adheres to our policy

All source data are provided with this paper in the Source Data file and the Github repository [https://github.com/JaegerLab/JEDI-1P_Widefiled_Figures_data doi:10.5281/zenodo.8209969]. The sequence of JEDI-1P is available from GenBank (accession number OP342601 [https://ncbi.nlm.nih.gov/nuccore/2476883238]). Plasmids used for in vitro characterization and packaging AAV for in vivo voltage imaging are available from addgene (accession numbers are pc3-puro-CAG-JEDI-1P-

P2A-mCherry-CAAX, 202606 [https://www.addgene.org/202606/]; pc3-puro-CAG-JEDI-1P, 202607 [https://www.addgene.org/202607/]; pAAV-EF1a-DIO-JEDI-1P-Kv, 202608 [https://www.addgene.org/202608/]; pAAV-EF1a-DIO-mCherry, 202620 [https://www.addgene.org/202620/]; pAAV-EF1a-DIO-tdTomato, 202610 [https://www.addgene.org/202610/]).

Human research participants

Policy information about studies involving human research participants and Sex and Gender in Research.

Reporting on sex and gender	N/A
Population characteristics	N/A
Recruitment	N/A
Ethics oversight	N/A

Note that full information on the approval of the study protocol must also be provided in the manuscript.

Field-specific reporting

Please select the one below that is the best fit for your research. If you are not sure, read the appropriate sections before making your selection.

☒ Life sciences ☐ Behavioural & social sciences ☐ Ecological, evolutionary & environmental sciences

For a reference copy of the document with all sections, see nature.com/documents/nr-reporting-summary-flat.pdf

Life sciences study design

All studies must disclose on these points even when the disclosure is negative.

Sample size	Sample sizes were set based on experimental variability observed in published work with similar experiments (e.g., https://doi.org/10.1016/j.cell.2022.07.013), as recommended by the NIH guidelines.
Data exclusions	For 96-well-based photostability characterization, wells with final masks selecting no more than 10000 pixels in a 512x512 FOV (under 1P) or no more than 100 pixels in a 512x32 FOV (under 2P) were excluded from the analysis as they are likely from low efficiency transfection. HEK293A cells were selected for patching if expression of the fluorescent indicators was detected; non-fluorescent cells were not chosen as they likely correspond to untransfected cells and no data can be acquired if cells are not fluorescent. We excluded patched cells from analysis if they did not pass our membrane test quality control (an access resistance (Ra) smaller than 12 MΩ and a membrane resistance (Rm) larger than 10 times Ra both before and after the recording). For 1P spectra characterization, spectra with excitation efficiency beyond ± 10% excitation efficiency at 350 or 535 nm were excluded with risk of improper autofluorescence correction. For simultaneous wide-field imaging and LFP recording, LFP recording with artifacts caused by respiration or motion that obscured the LFP signals were excluded prior to further analysis (LFP trials with artifacts were identified by amplitudes exceeding the normal range of LFP signals). For the analysis of photostability in vivo over 100 trials, 3 sessions were excluded because a full 100 trials were not available.
Replication	The value and definition of the sample size is provided in figure captions. For example, for field stimulation experiments, biological replicates correspond to individually transfected wells of a 96-well plate, and each data point in the paper represent a repeat of n >=4 wells. For patch clamp experiments, biological replicates correspond to individual cells, and each data point in the paper represent a repeat of n = 3-11 cells. For in vivo experiments, see figure captions.
Randomization	EMX1-Cre mice from the same colony were randomly selected for wide-field imaging experiments including JEDI-expression and their controls without JEDI AAV injections. This resulted in all non-injected control mice (N=4) being female. For other experiments (all in vitro characterizations), allocation was both random (cells were seeded randomly) and not relevant (the same cell line were used to compare between all indicators).
Blinding	No blinding was used, but all data were processed by automated scripts that are not biased towards any recording.

Reporting for specific materials, systems and methods

We require information from authors about some types of materials, experimental systems and methods used in many studies. Here, indicate whether each material, system or method listed is relevant to your study. If you are not sure if a list item applies to your research, read the appropriate section before selecting a response.

Materials & experimental systems

n/a	Involved in the study	
☒	☐	Antibodies
☐	☒	Eukaryotic cell lines
☒	☐	Palaeontology and archaeology
☐	☒	Animals and other organisms
☒	☐	Clinical data
☒	☐	Dual use research of concern

Methods

n/a	Involved in the study	
☒	☐	ChIP-seq
☒	☐	Flow cytometry
☒	☐	MRI-based neuroimaging

Eukaryotic cell lines

Policy information about cell lines and Sex and Gender in Research

Cell line source(s)	The HEK293A cell line was obtained from Thermo Fisher. The HEK293-Kir2.1 cell line was a gift from Gui-Rong Li Lab, who originally made this cell line; there is no commercial resource for this cell line.
Authentication	The HEK293A cell line and HEK293-Kir2.1 cell line were authenticated by STR profiling at MDACC.
Mycoplasma contamination	The HEK293A and HEK293-Kir2.1 cell lines were tested negative for micoplasma contamination by PCR tests.
Commonly misidentified lines (See ICLAC register)	No commonly misidentified lines were used.

Animals and other research organisms

Policy information about studies involving animals; ARRIVE guidelines recommended for reporting animal research, and Sex and Gender in Research

Laboratory animals	EMX1-Cre mice (JAX Strain 005628) were bred in house at Emory University. Adult mice (> 12 week old) were used for imaging experiments. Mice older than 4 weeks were used for histology. Mice were maintained at 72°F (22°C) with the humidity ranging from 30 to 70%.
Wild animals	No wild animals were used.
Reporting on sex	14 male and 15 female mice were used for all experiments. Of these 12 males and 11 females were used for in vivo JEDI-1P imaging. Sex-based analyses were not performed because no sex differences in voltage imaging quality were apparent from individual mice.
Field-collected samples	No field-collected samples were used.
Ethics oversight	All procedures to maintain and use mice were approved by the Institutional Animal Care and Use Committee at Emory University.

Note that full information on the approval of the study protocol must also be provided in the manuscript.
